# Draft genome of *Castanopsis chinensis*, a dominant species safeguarding biodiversity in subtropical broadleaved evergreen forests

**DOI:** 10.1186/s12863-023-01183-w

**Published:** 2023-12-14

**Authors:** Pan Chen, Ju-Yu Lian, Bin Wu, Hong-Lin Cao, Zhi-Hong Li, Zheng-Feng Wang

**Affiliations:** 1 Guangdong Forestry Survey and Planning Institute, Guangzhou, 510520 China; 2 Guangdong Provincial Key Laboratory of Applied Botany, South China Botanical Garden, Chinese Academy of Sciences, Guangzhou, 510650 China; 3 Key Laboratory of Vegetation Restoration and Management of Degraded Ecosystems, South China Botanical Garden, Chinese Academy of Sciences, Guangzhou, 510650 China; 4 South China National Botanical Garden, Guangzhou, 510650 China

**Keywords:** Gene structures, Genome feature, Genome annotation, Repetitive elements

## Abstract

**Objectives:**

*Castanopsis* is the third largest genus in the Fagaceae family and is essentially tropical or subtropical in origin. The species in this genus are mainly canopy-dominant trees, and the key components of evergreen broadleaved forests play a crucial role in the maintenance of local biodiversity. *Castanopsis chinensis*, distributed from South China to Vietnam, is a representative species. It currently suffers from a high disturbance of human activity and climate change. Here, we present its assembled genome to facilitate its preliminary conservation and breeding on the genome level.

**Data description:**

The *C*. *chinensis* genome was assembled and annotated by Nanopore and MGI whole-genome sequencing and RNA-seq reads using leaf tissues. The assembly was 888,699,661 bp in length, consisting of 133 contigs and a contig N50 of 23,395,510 bp. A completeness assessment of the assembly with Benchmarking Universal Single-Copy Orthologs (BUSCO) indicated a score of 98.3%. Repetitive elements comprised 471,006,885 bp, accounting for 55.9% of the assembled sequences. A total of 51,406 genes that coded for 54,310 proteins were predicted. Multiple databases were used to functionally annotate the protein sequences.

## Objective

*Castanopsis* is the third largest genus in the Fagaceae family [1]. It includes about 120–130 species in the genus [1–3]. Fossil evidence indicates that *Castanopsis* is widely distributed in both the Northern and Southern Hemisphere through the Eocene to the Pliocene in history [2, 4], but currently, it is mainly distributed in the subtropics and tropics of East and Southeast Asia [2, 4]. *Castanopsis* species are mainly canopy-dominant trees and can grow up to 25–40 m in height [5]. Therefore, they are the main components of evergreen broadleaved forests, safeguarding local biodiversity [2, 6]. *Castanopsis* species are good timber trees, and their seeds are edible [2, 3, 7]. They also contain many polyphenols and are used in traditional medicines [3]. Climate change is the main threat to *Castanopsis* species due to its restricted migration ability [3, 7]. By stud**y**ing 32 dominant *Castanopsis* species in East Asia that grow from 5°N to 38°N, it has been predicted that their present high richness distribution range will be reduced by 94.5%, on average, by 2070 [3].

China is a central region of the *Castanopsis* distribution and includes approximately 60 species [3], half of which are endemic to China [1]. *Castanopsis chinensis* is distributed from South China to Vietnam. It is a pioneer-dominant canopy tree in evergreen broad-leaved forests and plays a key role in ecosystems [3, 8]. As a fast-growing and soil erosion-controlling species, it is also widely used in reforestation [7]. Because the *C*. *chinensis* distribution area suffers from disturbances of high human activity, most forests have been converted or degraded. Therefore, we present here the *C*. *chinensis* genome to better understand its evolution and adaptation and enhance its conservation, management and utility in the future.

## Data description

We collected leaf samples of *C*. *chinensis* from a individual planted in the South China Botanical Garden, Guangzhou China. To perform genome assembly and annotation, three sequencing libraries were constructed from genomic DNA or RNA extracted from the leaf tissues. The first library was constructed by long read whole genome sequencing using a Nanopore PromethION sequencer, which generated about 139.5 GB of data (Data files 1–3) [9–11]. The second was generated by short read whole genome sequencing using an MGI DNBSEQ-T7 sequencer, which generated about 149.6 GB data (Data file 4) [12], and the third was generated by RNA sequencing (RNA-seq) using an MGI DNBSEQ-T7 sequencer, which generated about 29.7 GB data (Data file 5) [13]. All sequencing using the MGI platform was applied using 150 bp paired-end mode.

The genome size of *C*. *chinensis* was estimated by KmerGenie v1.7044 [14] (under the parameter of “-k 141 --diploid”) and GenomeScope 2.0 [15] (under the *k*-mer of 21) with short whole genome reads, which were trimmed using Sickle v1.33 [16] with the parameter “-q 30 -l 80”. The genome sizes estimated by KmerGenie and GenomeScope were 1,143,475,699 and 744,772,109 bp, respectively. Nanopore long reads were quality trimmed by Porchop v0.2.4 [17] and ontbc v1.1 [18] to remove adapters, and the reads had quality scores < 7 and lengths < 5000 bp. NextDenovo v2.3.1 [19] was then used to assemble the genome with the filtered reads. Pseudohaploid [20] and Purge_Dups v1.2.6 [21] (running twice) were used to remove duplicated contigs. Racon v1.5.0 [22], hapo-G v1.3.2 [23] and polypolish v0.5.0 [24] were further used to improve the assembly, in which racon and hapo-G were each run twice. The final assembly consisted of 133 contigs of 888,699,661 bp in length and a contig N50 of 23,395,510 bp (Data file 6) [25]. BUSCO v5.4.6 [26] assessed 98.3% completeness using the Eudicots odb10-2020-09-10 database (Data file 7) [27].

RED v2.0 [28] and EDTA v2.1.0 [29] were used to predict repetitive elements in the assembly, which revealed 400,198,509 (Data file 8) [30] and 410,582,904 bp (Data file 9) [31] of the sequences, respectively. Combining the RED and EDTA results resulted in 496,557,194 bp sequences (Data file 10) [32], accounting for 55.9% of the genome assembly. After soft-masking the repetitive elements in the assembly, braker v.2.0 [33] was used to predict the gene structures. Braker is an automated gene annotation pipeline that uses transcriptome data and reference protein sequences (Data file 11) [34]. The braker results were then inputted into Funannotate pipeline v1.8.16 [35] to obtain integrated gene sets. The pipeline included “train”, “predict” and “update” steps. In all steps, the parameter of “--max_intronlen 1000000” was used. In the “predict” step, the parameters of “--busco_seed_species arabidopsis --organism other --busco_db embryophyta” were added. The Funannotate pipeline finally produced 51,406 genes that coded 54,310 protein sequences (Data files 12–14) [36–38]. After gene structure prediction, functional annotation of the genes was performed using the “funannotate annotate” command in the Funannotate pipeline (Data files 15–16) [39, 40].

## Limitations

Although the genome size estimators of KmerGenie and GenomeScope were highly discrepant, yielding 1,143,475,699 and 744,772,109 bp, the final assembly size of 888,699,661 bp was comparable to previously reported genome sizes for *Castanopsis* species, including 878.6 Mb for *C*. *tibetana* [1] and 882.6 Mb for *C*. *hystrix* [41], and higher than 785.5 Mb for *C*. *mollissima* [42]. It has been reported that accurate genome size estimation with short reads is challenging [43, 44]. Therefore, long HiFi sequencing data may be further needed to obtain an accurate size estimation [44]. Due to their long length and high accuracy, HiFi date could character genome size reliably both in small and large *k*-mers [43, 45–48], which help determining the true result when the discrepancy happening in genome size estimation by short reads [43].

Currently, the assembled genome in this report is still fragmented. Therefore, it is not suitable for complete genome structure analysis, hindering the complex regions digging in its conservation and breeding [49–51]. Further high-quality genome assemblies (preferably complete and gapless) using ultra-long read, Hi-C, and other sequencing technologies are needed [52].

**Table 1:** Overview of all data files/data sets

Label	Name of data file/data set	File types (file extension)	Data repository and identifier (DOI or accession number)
Data file 1	Raw long whole genome sequencing reads	Fastq file (.fastq)	NCBI Sequence Read Archive, https://identifiers.org/ncbi/insdc.sra:SRR26081294 [9]
Data file 2	Raw long whole genome sequencing reads	Fastq file (.fastq)	NCBI Sequence Read Archive, https://identifiers.org/ncbi/insdc.sra:SRR26081295 [10]
Data file 3	Raw long whole genome sequencing reads	Fastq file (.fastq)	NCBI Sequence Read Archive, https://identifiers.org/ncbi/insdc.sra:SRR26081296 [11]
Data file 4	Raw short whole genome sequencing reads	Fastq file (.fastq)	NCBI Sequence Read Archive, https://identifiers.org/ncbi/insdc.sra:SRR26081292 [12]
Data file 5	Raw RNA reads of leaf tissues	Fastq file (.fastq)	NCBI Sequence Read Archive, https://identifiers.org/ncbi/insdc.sra:SRR26075029 [13]
Data file 6	Assembled genome	Fasta file (.fasta)	NCBI Nucleotide, https://identifiers.org/nucleotide:JAVQMG000000000.1 [25]
Data file 7	BUSCO assessment of the assembly	Text (.txt)	Figshare, 10.6084/m9.figshare.24417850.v2 [27]
Data file 8	Repetitive sequences predicted by RED	Text file (.bed)	Figshare, 10.6084/m9.figshare.24417889.v1 [30]
Data file 9	Repetitive sequences predicted by EDTA	Gff3 file (.gff3)	Figshare, 10.6084/m9.figshare.24417895.v1 [31]
Data file 10	Repetitive sequences combined by RED and EDTA	Text file (.bed)	Figshare, 10.6084/m9.figshare.24417910.v1 [32]
Data file 11	Table 1 Species with their protein sequences used for gene prediction	Table (.xlsx)	Figshare, 10.6084/m9.figshare.24417970.v1 [34]
Data file 12	Predicted gene	Gff3 file (.gff3)	Figshare, 10.6084/m9.figshare.24417985.v1 [36]
Data file 13	Predicted genes - nucleotide sequences	Fasta file (.fasta)	Figshare, 10.6084/m9.figshare.24417991.v1 [37]
Data file 14	Predicted genes - translated sequences	Fasta file (.fasta)	Figshare, 10.6084/m9.figshare.24418003.v1 [38]
Data file 15	Gene annotation using GO, Pfam, interPro, UniProt, dbCAN, MEROPS and SignalP databases	Text (.txt)	Figshare, 10.6084/m9.figshare.24418012.v1 [39]
Data file 16	Gene annotation from eggNOG-mapper analysis	Text (.txt)	Figshare, 10.6084/m9.figshare.24418015.v1 [40]

## Data Availability

Raw sequenced reads have been uploaded to the NCBI Sequence Read Archive under accession number SRR26081294, SRR26081295 and SRR26081296 for long whole genome sequencing reads [9–11], SRR26081292 for short whole genome sequencing reads [12], SRR26075029 for RNA-seq reads [13], and JAVQMG000000000 for the assembled genome [25]. Please further see Table 1 for details and references [30–32, 34, 36–40] of the results of the annotations submitted to figshare.
